# Troubled social background of male anabolic-androgenic steroid abusers in treatment

**DOI:** 10.1186/1747-597X-2-20

**Published:** 2007-07-05

**Authors:** Kurt Skarberg, Ingemar Engstrom

**Affiliations:** 1 Department of Clinical Medicine, Psychiatric Research Centre, Orebro University, Sweden; 2 Addiction Centre, Orebro County Council, Orebro, Sweden

## Abstract

**Background:**

The aim of this study was to investigate the social background and current social situation of male abusers of anabolic-androgenic steroids (AAS).

**Methods:**

We compared thirty-four AAS-abusing patients from an Addiction Centre (AC) with two groups, 18 users and 259 non-users of AAS from a public gym in Orebro, Sweden. The study is based on semi-structured interviews and questionnaires.

**Results:**

Histories of a troubled childhood as well as current social disadvantage were both more frequent among the AAS users. Users also reported poor relationships with their parents and almost half of them had experienced physical or mental abuse. The AC group's experiences from school were mostly negative, and included concentration problems, boredom and learning difficulties. Their current circumstance included abuse of other drugs, battering of spouses and other criminality such as assault, illegal possession of weapons and theft.

**Conclusion:**

In conclusion, this study shows that abusers of AAS often have a troubled social background. This underlines the importance of making a thorough social assessment as a part of the treatment programme. The results of the study may help in directing appropriate questions relevant to the abuse of AAS.

## Background

Experimenting with anabolic-androgenic steroids (AAS) has been common among athletes for several decades [1]. In the 1950s and 1960s, usage was almost entirely restricted to highly trained athletes [2]. Later, AAS abuse spread from professional to high school sports performers [3] and these substances are now also being abused by those whose training is purely recreational and/or cosmetic [4].

Today, the abuse of AAS is thus no longer confined to elite athletes but can be found among various groups of people. Until recently, AAS abusers were rarely seen at addiction clinics in Sweden, but today they have begun seeking help there. Unfortunately, even clinicians working at addiction clinics frequently neglect to ask about AAS in their history taking [5]. It has therefore become important to encourage the systematic gathering of information about the lives and backgrounds of these patients, so that effective treatment programmes may be designed.

The abuse of AAS is often combined with use of other hormones such as growth hormone (GH), insulin, thyroid hormone, insulin-like growth factor 1 (IGF 1) [6], other doping agents such as Clenbuterol, Ephedrine, Tamoxifen, Gammahydroxybutyrate (GHB) [7] and other drugs [8,9]. These doping agents are also known as "body image" drugs [10].

AAS may cause medical and psychiatric illness [11-13], including dependence syndromes [7], psychological dependence [14] or withdrawal symptoms, such as AAS craving [7]. AAS may also stimulate aggressive behaviour, criminal activity, violence [15,16], and suicide and homicide [17]. These side-effects may also put those who come into contact with abusers at risk [18]. It is therefore imperative that we enhance current knowledge about AAS abusers.

To our knowledge, no specific studies of the social backgrounds of AAS abusers have previously been carried out. Some studies, however, provide information about risk factors for AAS abuse, which may be of a social nature.

There is consensus that AAS use is more common among males than females [9,19-21]. It has been noted that AAS abusers often have poor relationships with their fathers [22]. Other risk factors that have been reported are unsupervised recreation, poor social support [4] and a clear avoidance of social contacts [23].

The significance of peer pressure as a trigger for AAS abuse has been emphasized by many researchers [14,24-26]. Seeing friends suddenly grow and gain bulk may encourage others in a group to try AAS [25]. A Swedish study [27] found that AAS abusers reported poorer relationships with their peers and lower levels of academic achievement than non-users. Other risk factors may be dissatisfaction with school, living alone at an early age, repeated truancy from school and frequent strength training [9].

A history of behavioural disorders in childhood is often reported by AAS abusers [22] as is hyperactivity [18]. Abusers have also been shown to have low self-esteem [16,27] and/or self-confidence [22].

Our survey of the literature found no specific studies of the social backgrounds of AAS abusers. The available information is mostly derived from questionnaires used in high schools, while we have found none that examines the social circumstances of those who attend addiction clinics. We therefore consider it highly relevant to explore this question from a clinical perspective.

The aim of this study was to describe the social background and current social situation of AAS abusers who were seeking treatment at an addiction centre and to compare the findings with findings from gym clients with and without a history of AAS abuse. We propose that knowledge in this area is of importance for the appropriate design of treatment programmes for AAS abusers.

## Methods

### Selection of subjects

The male AAS-abusers were consecutively included under three years from a psychiatric addiction clinic (AC) (Beroendecentrum) in Orebro county, central Sweden, a county of 275 000 inhabitants. The inclusion criteria for patients were that they must: a) be over 16 years of age, b) be fluent in Swedish, c) be using non-prescribed AAS, alone or in combination with other doping agents, d) have been using AAS for at least four consecutive months and e) be under care in the addiction clinic where a decision to commence treatment had been agreed upon following with an intention to treat, based on the initial clinical assessment. The AC AAS abusing group, from now on called AC group, consisted of 34 male patients.

The comparison groups were recruited from gym clients in Orebro. These groups were chosen because all individuals in the AC group were gym clients, a finding that has been noted in earlier studies [22]. Participants for the study were sought by putting up posters at the gym. Two hundred eighty nine males responded to the questionnaire. 12 people, who did not answer the questions about hormones, were excluded from the study. Those who then remained, 277 people, were divided into two comparison groups: 18 male gym clients who had used AAS at some time and 259 male gym clients who had not used AAS at any time, according to their self-report. Both of these groups fell into the same age range as the AAS group (18–45 years).

### Questionnaire

The AC group were interviewed using a semi-structured format that was based on a clinical interview structure that had been used at the clinic for several years for investigating social factors. The interview consisted of questions concerning the person's social background and current social situation. The questions about social background covered family history, contact with parents and other relatives, physical or mental abuse, school experiences, education, vocational training, criminality, drug use history and relations with partners. Questions about the person's current social situation concerned their housing, occupation, ongoing physical training and current use of alcohol and drugs.

For our comparison groups we designed a 50-item questionnaire, based wholly on the interview format described above. The questions were kept as close as possible to the interview schema. The answer options were derived from the answers we received in the interviews with the addition of a few extra, open alternatives. The questions concerning alcohol consumption were taken from AUDIT (The Alcohol Use Disorders Identification Test), which was developed by the World Health Organization [28] and which has been translated into Swedish [29]. The questionnaire was distributed through the reception at the gym. Respondents left their questionnaires anonymously in sealed envelopes dropped into a box.

### Dropout analysis

No one in the AC group declined to participate in the study. The results from the 12 potential participants in the gym who were excluded due to a history of AAS abuse will, however, be analysed separately.

### Ethical approval

The study protocol was approved by the Research Ethics committee of Orebro County Council, # 538/99.

### Statistics

Statistical analysis of numerical data was carried out using a one-way ANOVA for equality of means and two-sided Fisher's exact test for comparison of three groups. The SPSS software package version 14.0 was used. A significance level of p < 0,05 was considered appropriate.

## Results

### Age and biological data

The mean age of the AC group was 27.2 years, for the non-users 26.1 years and for the gym AAS-abusers 34.8 years (all in the range 18–45). The mean age of initial use of AAS in the AC-group was 20.7 years (range 15–30) (not known in the gym AAS-abuser group).

Table 1 shows that the groups differed significantly, with respect to weight and BMI. The AAS-abusers from the gym were on average older and shorter than the other groups. The AC group tended to be heavier than the other groups.

**Table 1:** Comparison of basic biological data between AAS users in two groups and non-users.

	AC group, n = 34		Gym, AAS-users, n = 18		Gym, non-users, n = 259		p-value ^1^
	Mean	SD	Mean	SD	Mean	SD	

Age (years)	27.2	5.7	34.8	8.4	26.1	7.0	<0.001
Height (cm)	179.0	6.2	177.8	5.4	181.1	6.6	<0.05
Weight (kg)	100.4	18.4	94.3	13.5	83.2	12.4	<0.001
BMI^2^	31.12	5.04	29.73	3.42	25.32	3.31	<0.001
First used AAS (years)	20.69	4.03	-	-	-	-	

^1 ^Oneway ANOVA for equality of means (df 2, 308).

^2 ^Body Mass Index (kg/m^2^)

### Social background

The social background variables are presented in table 2. Almost all of the participants were born in Sweden (in total 93 %) and 79 % came from families in which both parents had been born in Sweden. There were no significant differences between the groups in this respect. Sixty-two percent of the AC group grew up with both parents, while 67 % of the gym AAS-abusers and 79 % of the non-using group did so. Growing up with only their mother or with someone other than one's own parents (usually grandparents) was thus more common among members of the AAS groups compared to the non-user group.

**Table 2:** Comparison of social background between AAS users in two groups and non-users.

		AC group, n = 34		Gym, AAS-users, n = 18		Gym, non-users, n = 259		p-value ^1^
		%	n	%	n	%	n	

Born in Sweden	Yes	97.1	33	88.9	16	92.7	240	0.475
	No	2.9	1	11.1	2	7.3	19	
Parents born in Sweden	Both	88.3	30	61.1	11	79.5	206	0.187
	One	8.8	3	22.2	4	11.2	29	
	None	2.9	1	16.7	3	9.3	24	
Brought up with....	Both biological parents	62.5	20	66.7	12	78.9	203	<0.05
	Mother only	28.1	9	27.8	5	18.7	48	
	Father only	0.0	0	0.0	0	1.2	3	
	Other	9.4	3	5.6	1	1.2	3	
Quality of upbringing	Good	43.8	14	72.2	13	87.2	224	<0.001
	Indifferent	15.6	5	5.6	1	10.1	26	
	Bad	40.6	13	22.2	4	2.7	7	
Single child	Yes	27.3	9	35.3	6	13.9	36	<0.05
	No	72.7	24	64.7	11	86.1	223	
Divorced parents	Yes	59.4	19	44.4	8	38.3	98	0.071
	No	40.6	13	55.6	10	61.7	158	
Age at the time of divorce (years)	0–6	5.9	1	50.0	4	37.0	34	<0.05
	7–12	41.2	7	12.5	1	20.6	19	
	>12	52.9	9	37.5	3	42.4	39	
Relation with mother	Good	68.7	22	88.9	16	93.7	239	<0.001
	Indifferent	21.9	7	5.6	1	5.5	14	
	Bad	9.4	3	5.6	1	0.8	2	
Relation with father	Good	46.9	15	61.1	11	76.1	194	0.001
	Indifferent	28.1	9	5.6	1	8.6	22	
	Bad	25.0	8	33.3	6	15.3	39	
Other significant person	Yes	54.5	18	58.8	10	65.4	168	0.401
	No	45.5	15	41.2	7	34.6	89	
Physically abused	Yes	30.3	10	22.2	4	5.8	15	0.001
	No	69.7	23	77.8	14	94.2	242	
Mentally abused	Yes	48.5	16	27.8	5	10.2	26	<0.001
	No	51.5	17	72.2	13	89.8	230	
Age at the time of moving away from home (years)	Not yet moved	7.1	2	0.0	0	14.4	36	0.090
	11–15	10.7	3	0.0	0	2.4	6	
	16–20	60.7	17	82.4	14	52.0	130	
	21–25	21.5	6	17.6	3	30.8	77	
	>25	0.0	0	0.0	0	0.4	1	
Drug abuse in the family	Yes	43.7	14	35.3	6	19.9	51	<0.01
	No	56.3	18	64.7	11	80.1	205	
Criminality in the family	Yes	18.8	6	27.8	5	12.1	31	0.097
	No	81.2	26	72.2	13	87.9	225	

^1 ^Fisher's exact test, 2-sided for three groups

The majority of the members of the AC group had divorced parents. The divorce had generally taken place later in the AC group than in the comparison groups; only six percent had divorced before the child had started school while the corresponding figure for the gym group was 50 % and for the non-using group, 37 %.

The groups differed significantly in how they evaluated their relationships with their parents. When they were asked to qualify their contact with their mother and father respectively as either "good", "bad" or "indifferent" the vast majority of the members of the non-using group described their relationship with their mother as good. Least good relationship with their mother reported the AC group. The same result were found with regard to the relation with their father. Best relations were found among the members of the non-using group and least good in the AC group.

We asked the participants if there had been any significant other person available to them when they were children. The responses we received from members of the three groups showed no significant differences. However, all three groups noted grandfathers as the most significant other persons in their lives: sixty-eight percent of the non-using group, 50 % of gym AAS-abusers and 23 % of the AC group.

The AAS-abusers had experienced significantly more physical and mental abuse than the non-using group. The perpetrator of the physical abuse of the AC group was usually the father (60 %), and/or another relative (40 %), the mother (20 %) or siblings (20 %). In the gym AAS-abuser group the perpetrator was often the mother (75 %) and/or the father (50 %). It was more common to find a history of mental abuse among the AAS abusers groups compared to the non-using group. As with physical abuse, the perpetrator for the AC group was most commonly the father (87 %), and/or the mother (69 %), another relative (31 %) or siblings (12 %). In the gym AAS-abuser group the perpetrator was often the father (60 %), and/or another relative (40 %) and/or the mother (20 %).

Drug abuse in the family was significantly more common in the AC group compared to the non-user group. This was true both for alcohol (Fisher's exact p < 0.01) and for pharmaceuticals (Fisher's exact p < 0.01) but not for illegal drugs (Fisher's exact p = 0.265). The incidence of criminality in the families of the three groups did not differ significantly.

### Education and school problems

The educational backgrounds of the three groups are reported in table 3. In general, the members of the non-using group had a higher educational level than those of the AAS groups. Over 33 % of the gym AAS-abusers and almost a quarter of the AC group had only completed primary schooling.

**Table 3:** Comparison of education and school problems between AAS users in two groups and non-users.

		AC group, n = 34		Gym, AAS-users, n = 18		Gym, non-users, n = 259		p-value ^1^
		%	n	%	n	%	n	

Educational level	Interrupted primary school	0.0	0	0.0	0	3.9	10	<0.01
	Primary school	24.2	8	33.3	6	8.9	23	
	High school	69.7	23	55.6	10	63.2	163	
	Higher education	6.1	2	11.1	2	24.0	62	
Well-being in school	Yes	18.7	6	50.0	9	74.7	192	<0.001
	Indifferent	21.9	7	33.3	6	15.6	40	
	No	59.4	19	16.7	3	9.7	25	
School truancy at least once a week	Yes	62.5	20	11.1	2	17.5	45	<0.001
	No	37.5	12	88.9	16	82.5	212	
Bullied others in school	Yes	25.0	8	61.1	11	31.0	80	<0.05
	No	75.0	24	38.9	7	69.0	178	
Been bullied in school	Yes	43.8	14	44.4	8	27.4	71	0.069
	No	56.2	18	55.6	10	72.6	188	
Academic difficulties	Yes	93.7	30	38.9	7	25.6	66	<0.001
	No	6.3	2	61.1	11	74.4	192	
...as concentration problems	Yes	78.1	25	27.8	5	12.5	32	<0.001
	No	21.9	7	72.2	13	87.5	223	
...as writing problems	Yes	21.9	7	27.8	5	7.1	18	<0.01
	No	78.1	25	72.2	13	92.9	236	
...as reading problems	Yes	9.4	3	27.8	5	6.7	17	0.010
	No	90.6	29	72.2	13	93.3	238	
...as boredom	Yes	50.0	16	22.2	4	13.7	35	<0.001
	No	50.0	16	77.8	14	86.3	220	
...as drug abuse	Yes	37.5	12	22.2	4	5.9	15	<0.001
	No	62.5	20	77.8	14	94.1	240	
...as mental problems	Yes	12.5	4	0.0	0	1.6	4	0.010
	No	87.5	28	100.0	18	98.4	251	

^1 ^Fisher's exact test, 2-sided for three groups

It is clear that the vast majority of the AC group did not have a positive experience of their school years and over 62 % of them reported frequent school trauncy. However, we found no significant differences concerning experiences of having been bullied in school but the gym AAS-abuser group reported a significantly higher rate of having bullied others in school.

Thirty of the thirty-four members of the AC group reported having had academic problems in school. This represents a highly significant difference from the comparison groups. The reasons for the reported 'problems at school' were several e.g. concentration problems, boredom, drug abuse and mental problems. All of these were significantly more common in the AC group, particularly concentration problems, boredom and drug abuse, compared to the gym groups. The AAS-abusers had more specific writing problems and the gym-abusing group had significantly more reading problems.

### Current social situation

The current social situation of the groups is tabulated in table 4. Housing conditions differed significantly between the groups. Almost nine percent in the AC group had no housing and the majority lived alone. Some of the men who were living alone had their rent paid by their mothers and many would either eat and/or sleep at their mother's house. Stable relationships with partners were significantly more common in the gym AAS-abuser group than the other groups. It was more common among both AAS abuser groups to have children compared to the non-user group.

**Table 4:** Comparison of current social situation between AAS users in two groups and non-users.

		AC group, n = 34		Gym, AAS-users, n = 18		Gym, non-users, n = 259		p-value ^1^
		%	n	%	n	%	n	

Housing	Homeless	8.8	3	0.0	0	0.0	0	<0.001
	Living alone	52.9	18	18.8	3	40.2	102	
	Living with partner	5.9	2	0.0	0	16.5	42	
	Living with partner	26.5	9	75.0	12	37.8	96	
	Other	5.9	2	6.3	1	5.5	14	
Established partner	Yes	44.1	15	88.9	16	53.8	136	<0.01
	No	55.9	19	11.1	2	46.2	117	
Children	Yes	36.4	12	41.2	7	14.7	38	0.001
	No	63.6	21	58.8	10	85.3	221	
Threatened partner	Yes	39.4	13	22.2	4	8.6	22	<0.001
	No	60.6	20	77.8	14	91.4	235	
Battered partner	Yes	30.3	10	11.1	2	6.6	17	<0.001
	No	69.7	23	88.9	16	93.4	240	
Income from	Work	55.9	19	77.8	14	86.1	223	<0.001
	Subsidiary	38.2	13	16.7	3	7.3	19	
	Other	5.9	2	5.6	1	6.6	17	
Alcohol	Risk consumption or abuse	52.9	18	11.1	2	17.9	46	<0.001
	Regular or no	47.1	16	88.9	16	82.1	211	
Narcotics	Yes	91.2	31	61.1	11	26.6	69	<0.001
	No	8.8	3	38.9	7	73.4	190	
Unprescribed pharmaceuticals	Yes	57.6	19	41.2	7	11.8	30	<0.001
	No	42.4	14	58.8	10	88.2	225	
Driving license	Yes	47.1	18	72.2	13	77.0	194	<0.05
	No	52.9	16	27.8	5	23.0	58	
Sentenced for crime	Yes	97.1	33	38.9	7	16.5	42	<0.001
	No	2.9	1	61.1	11	83.5	213	
Food additives	Yes	90.6	29	88.9	16	75.2	194	0.075
	No	9.4	3	11.1	2	24.8	64	
Training frequency per week	>4 times	35.3	12	16.7	3	16.2	42	0.096
	3–4 times	47.1	16	72.2	13	67.2	174	
	<3 times	17.6	6	11.1	2	16.6	43	

^1 ^Fisher's exact test, 2-sided for three groups

It was more common that members of the AC group at some time assaulted and/or threatened their female partners compared to those in the non-using group.

Approximately half of the AC group held steady employment and which was less than in the gym AAS-abusers and the non-using group. Thirty-eight percent of the AC group was living off social security or sickness benefits.

The AAS-abusers used alcohol, drugs and non-prescribed pharmaceuticals to a greater extent than members of the non-using group. In the AAS abuser groups it was more common that members of the AC group abused alcohol, narcotics and unprescribed pharmaceuticals compared to the gym AAS abusers group. Twenty-two percent of the gym AAS-abusers had sniffed solvents compared to 12 % of the AC group and almost nine percent of non-users (ns). Moreover, more than half of the AC group (53 %) consumed alcohol in quantities that would qualify as abuse according to AUDIT [28], while this was true of only 18 % of the non-using group and 11% of the gym-abusing group.

All individuals in the AC group except for one admitted having been found guilty of some kind of crime, while 39 % of gym AAS-abusers and only 16 % of the non-using group reported this. The most common kinds of crime in the AC group were assault (61 %), illegal drug use (54 %), illegal possession of weapons (42 %), burglary (39 %), theft (39 %) and selling doping agents, (39 %). According to gym AAS-abusers the most common crime among them was illegal drug use, for which 22 % had been found guilty.

All members of these three groups were training at gyms, though the members of the AC group did so slightly more frequently. The most common motivation for training at gym given by AC group was to improve physique (76 %), while 57 % of non-users and 59 % of gym AAS-abusers reported this. The desire to enhance achievement in sport was reported by 65 % of the AC group, compared to 22 % of the non-using group and none of the gym AAS-abusers. Only three percent of the AC group mentioned enhanced well being as a reason for training, while 83 % of the gym AAS-abuser group and 65 % of the non-users reported this as a motivation. Also of interest is the fact that only 22 % of gym AAS-abusers and 17 % of non-users claimed they trained for fun, while no one from the AC group did so.

## Discussion

The aim of this study was to examine the social background and current social situation of a group of AAS abusers who were patients at an addiction clinic in central Sweden. Two groups of people who also trained frequently at a gym, one group of AAS-abusers and one group that had no experience of AAS, were recruited for comparison. The gym AAS-abusers were generally older than the members of the other two groups but were still in the same age range, 18–45 years. We consider these three groups to be comparable since the members were of the same sex and had similar training habits.

The social backgrounds in the AC group were found to be relatively disadvantaged in comparison to the non-using group. The gym AAS-abusers fell between the other two groups in this regard, but they were often nearly as disadvantaged as the AC group members. The family backgrounds of the AAS-abusers were often problematic in a variety of ways. They had generally poor social support, their parents were often divorced, and in the AC group the divorce had usually taken place at a fairly late stage in the child's development. Together with other information about the use of drugs and history of physical and/or mental abuse in the family, these factors suggest that AAS-abusers had been brought up in families with a high degree of intra-familial conflict.

The AAS-abusers often described poor or indifferent relations with their parents. Less than half of the member of the AC group described their relationships with their fathers as positive. Like an earlier study [22], ours showed that AAS-abusers had poorer relationships with their fathers than non-users. The importance of fathers is well recognized so it was no surprise to find that these conflict-ridden families involved worse relationships between fathers and sons than between mothers and sons.

In both AAS-abuser groups, we found a common pattern of frequent physical and/or mental abuse, which reinforces the image of conflict-filled milieus. The father was the most common perpetrator, but in quite a few cases mothers were also responsible, particularly when it came to physical abuse in the gym AAS-abuser group.

Almost half of the AC group and one third of gym AAS-abusers came from families in which one or both of the parents were alcoholics or drug addicts, and this was often associated with various forms of criminality.

Just over half of the AAS-abusers could name a particular person who had played an important role as 'significant other' during their childhood. The most common "significant other" was a grandfather. The presence of a significant other is generally considered to provide a buffer for children in adversity [30]. It is especially notable that the AAS-abusers did not identify any friends whom they felt had been of great importance when they were children.

The general picture of the family backgrounds of both AAS-abusers groups is therefore somewhat bleak. The typical scenario consists of a conflict-ridden family in which the son feels alone, with no significant other to turn to, even among his friends.

Like Kindlundh et al[9], we found that AAS-abusers also encountered far greater difficulties at school than did their counterparts from the non-using group. Almost all in the AC group described their school experience as very negative in one or more respects. The AAS-abusers educational level was also generally lower than that of members of the non-using group. This is probably a result of the combination of adverse family conditions and negative experiences of school. The AAS-abusers described a variety of problems from their time at school. The most frequently reported problems in the AC group were lack of concentration and specific reading/writing difficulties, but also well-being in school, truancy and boredom. Both AAS-abusing groups reported having been bullied, experienced writing/reading problems and problems with drug abuse and/or mental problems of various kinds. The gym AAS-abusers, however, more frequently reported having bullied others in school than did the AC group and non-users.

The AC group in our study was often living alone or had no housing of their own. It is known from earlier studies that children who grow up with "risk" elements in the environment may get difficulties with intimate relations [30]. It is also known that AAS abuse often co-exists with abuse of other drugs [6]. Most of the AC group and many of the gym AAS-abusers were mixed drug abusers, and this may have had a negative impact on their social situation since mixed drug abusers often buy their drugs before paying their rent.

The AC group were less likely to be living with a stable partner than non-users, but both AAS-abuser groups had more children than non-users. AAS often enhances sex drive initially [31] and this may lead to abusers engaging in serial, short-lived sexual relationships. The frequency of physical and mental abuse of female partners is extremely high among AAS-abusers and this may be caused by the irritability that AAS induces.

The non-users in this study were more likely to have salaried employment than AC group, most of whom were dependent on social security and/or sickness benefits. The AC group were seldom able to hold a job for long and, if they were working at all, it was often in temporary employment. The difference found regarding possession of driving licenses might be due to the loss of driving privileges in patients who had higher rates of alcohol abuse and associated drunk-driving episodes.

It has been noted that AAS may provoke criminal activity and violence [16]. The AC group in our study reported an extremely high rate of criminality, which usually involved various kinds of violence. The design of this study did not enable us to examine the temporal relationship between AAS use, other drug use and criminality, but we are currently planning further investigations to explore these sequences of events in more detail.

A small group of 12 persons (with a mean age of 24.2 years) did not respond to the question regarding history of AAS abuse. When we analysed their answers they proved to fall somewhere between the two AAS-abuser groups. We suspect, however, that they should be considered AAS-abusers but since they did not complete the questionnaire we had to exclude them.

To our knowledge, this study is the first to systematically examine the social backgrounds of AAS-abusers. The size of the AAS groups was not large. It is not easy to recruit subjects to this kind of study since they are usually hesitant to admit their AAS use, to seek help or to remain under medical care when they begin to feel better. The findings from this study cannot therefore be generalized to all forms of AAS use since these particular groups were also involved with abuse of other drugs. However, in our experience, exclusive use of AAS is quite uncommon. Sooner or later, AAS-abusers tend to start using other drugs as well, primarily central stimulants. We are, however, aware of the fact that the study group in this case was involved in relatively serious drug abuse and this should be borne in mind when drawing conclusions.

We found a high frequency of reported use of narcotics in both AAS groups, which was surprising since patients seeking help may be expected to be more honest than non-patients about their use of other drugs. It is also of significance that the AC group, who want help with their drug problem, are subject to drug testing. However, the unexpectedly high rate of reporting of the use of narcotics in the gym AAS-abuser group and among non-users suggests that the methods used may be fairly reliable.

The statistical comparisons were predominantly done using Fisher's exact test for three groups, which show whether there was an overall statistically significant difference between the three groups. We have refrained from doing post-hoc tests between paired groups in order to keep the statistical methods conservative, not to draw too far-going conclusions. There would otherwise have been a risk for a statistical effect of multiple comparisons as well as statistical power problems due to small numbers in some of the group comparisons.

Another methodological issue that warrants comment is the fact that the AC-group was interviewed while the comparison groups simply answered a questionnaire. Our original intention was to recruit a clinical comparison group whose members were performing physical training and using drugs, but not AAS. However, this proved impossible since we were simply unable to find people that met these criteria, which leads us to conclude such people are rare. Our second option was therefore to recruit a large group of people who were training but who were not taking AAS. We found, though, that this group inevitably ended up containing both AAS-users and non-users.

The study was carried out using a mixture of methods; interviews in the index group and questionnaires in the comparison groups. This combination of methods must be taken into consideration when comparing the results. All of the interviews were carried out at the clinic by one experienced clinician in order to minimize the possibility of misunderstandings and varying interpretations of questions and answers. Anonymity was guaranteed for participants from all groups. The use of a questionnaire was decided upon for the larger comparison group for practical reasons. It meant we were able to obtain a much larger sample than would have been possible using interviews. In order to reach optimal comparability, the questions used in the questionnaire were derived from the responses received in the interviews [32]. Although questionnaires are known to make it easier for respondents to answer sensitive questions [33] the question concerning the use of doping agents was the one most frequently left unanswered. This means that the difference between the non-using and abusing-groups might be even greater than it would seem according to the responses, and this would support our contention that the reported differences are not overestimations.

## Conclusion

This study has shown that abusers of AAS often come from severely disadvantaged family backgrounds and that they also live their adult lives in difficult social situations. This study is based on a fairly small and selected sample and it is therefore not possible to extrapolate the results to AAS-abusers generally, but since there are very few studies of AAS-abusers in substance abuse treatment, we believe that the results are nevertheless of significant value. It is clearly of great clinical value to track the social backgrounds of AAS-abusers and to pay close attention to the conditions under which they are currently living. We propose that an interview that explores specific social issues should form an integral part of the treatment programme. The results of this study may help in directing appropriate questions relevant for AAS abuse, which we do not believe are being put forward in general clinical practive.

We intend to continue studying this group, particularly with regard to the relationship over time between factors such as alcohol abuse, AAS abuse, drug abuse and criminality. In this way we hope to enrich our understanding of the risks associated with AAS use, both from a social and from a medical perspective.

## Competing interests

The author(s) declare that they have no competing interests.

## Authors' contributions

KS conceived of the study, participated in its design, carried out all interviews and gathered the questionnaire data, performed the statistical analysis and drafted the manuscript. IE was responsible for the design and helped to draft the manuscript. Both authors read and approved the final manuscript.
